# Trifurcated lined ducts: A comprehensive study on noise reduction strategies

**DOI:** 10.1371/journal.pone.0306115

**Published:** 2024-07-26

**Authors:** Touqeer Nawaz, Muhammad Afzal

**Affiliations:** 1 Department of Mathematics, Capital University of Science and Technology, Islamabad, Pakistan; 2 Department of Mathematics and Natural Sciences, Center for Applied Mathematics and Bioinformatics, Gulf University for Science & Technology, Hawally, Kuwait; Universiti Brunei Darussalam, BRUNEI DARUSSALAM

## Abstract

The present research is centered on analyzing and modeling the scattering characteristics of a trifurcated waveguide that includes impedance discontinuities. A mode-matching method, grounded in projecting the solution onto orthogonal basis functions, is devised for the investigation. The impedance disparities at the interfaces are represented in normal velocity modes, which, when combined with pressure modes, result in a linear algebraic system. This system is subsequently truncated and inverted for numerical experimentation. The convergence of scattering amplitudes is assured by reconstructing matching conditions and adhering to conservation laws. The computational results indicate that optimizing attenuation behavior is achievable through manipulating variation bounding properties and impedance discontinuities.

## Introduction

The theory of noise reduction has become a dynamic area of research due to large-scale industrial advancements. This research is crucial for various applications, including, aircraft and vehicle engines, turbofan engines, ducts, and pipes. Guided wave systems, known for their efficiency in carrying acoustic energy by preventing lateral diffusion, resist the decay of sound waves according to the inverse square law.

Numerous scientists and engineers have addressed noise reduction by considering different material properties of ducts and diverse geometrical designs. Rawlins [1] discussed noise reduction through a duct with a thin acoustical absorbent lining on parallel plates partitioning. According to Demir and Buyukaksoy [2], fixing the walls of a conduit with an acoustically lining material can fundamentally improve its acoustic performance. Morse [3] investigated the attenuation of sound in boundless shut pipes using acoustically absorbing liners. Subsequent analyses confirmed that fixing the properties of on the walls of waveguide enhances sound absorption.

The study of waveguides based on different mathematical formulations has been extensively discussed. Koch [4] introduced the Wiener-Hopf solution to specify the problem of the radiation of sound from a semi-infinite 2D channel with walls fixed with a responding sound retention substance. Jones [5] evaluated the far field and near-zone solutions for the issue of wave dispersion from equally spacing walls. Jones’ analysis was further extended by Asghar et al. [6] to address the response of line and point sources in stationary compressible fluid. Hassan and Rawlins [7] then provided exact and closed-form structure solutions with parallel plate spacings to analyze the scattering characteristics of semi-endless soft-hard pipe or duct. Furthermore, the acoustic radiations caused by a uniform parallel plate for various medium and bounding material attributes have been thoroughly explored [8]. For instance, studies [9–11] incorporated soft, hard, or permeable linings, as well as fixed and fluid flow media in a waveguide containing trifurcation. These situations illustrate that the Fourier modification was still appropriate because the wave number observed a consistent range. They conducted a numerical evaluation investigate the reflection of fundamental mode, which requires challenging split functions and is important practically.

From the idea of orthogonality relations, the mode-matching solution framework is described for examining the scattering of waves in waveguides comprising discontinuous material properties and abrupt geometric changes. The discussion of the energy flux and power balance is followed by appropriate numerical experiments that clarify the findings. Recently, this technique is widely used in the scattering analysis of complex configurations, effectively managing the material and geometric discontinuities, for instance see Afzal et al [12–14] and Nawaz and Lawrie [15].

Many authors investigated the acoustic wave diffraction in a planar waveguide with various boundary parameters by using analytical techniques that rely on the consideration of continuous spectrum of wave numbers such as Wiener-Hopf technique, for instance see [16–19]. However, to the best of the authors’ knowledge no attempt has been made to consider step discontinuity in a more practical setting to study the planar/non-planar waveguide. The classical Wiener-Hopf technique fails to provide scattered fields for the more complex systems whereby the problem contains discontinuities in structures and/or material properties. Nevertheless, for the inhomogeneous and non-uniform waveguides, the improved multimodal method for the acoustic propagation in waveguides offers advancements in efficiently describing acoustic propagation, addressing low convergence issues, for instance [20–22]. However, many times this method may not be preferred due to the limitations on the number of modes involved in high-frequency computations. On the other hand, numerical methods such as the finite element method [23] or boundary element method [24, 25], it is possible to study a silencer of any shape or size. However, with increasing excitation frequency and silencer dimensions, the number of degrees of freedom in the problem increases rapidly, for instance see [23]. Nevertheless, the meshless wave-based method for modeling sound propagation is a cutting-edge approach that leverages the singular boundary method and Kansas method to accurately simulate acoustic wave propagation in heterogeneous media, providing an alternative to traditional mesh-based methods [26–28]. Nevertheless, the meshless wave-based method, while offering several advantages, may be limited to the problems involving discontinuities in geometrical and/or material properties because of numerical stability issues. Recently, the mode-matching method is applied to deal bifurcated and trifurcated waveguide problems, for instance see [29–31].

The article includes an investigation of the scattering characteristics of a rigid inlet duct placed in a lined duct connected to outlet lined duct via impedance discontinuities in a more general and practical context. The study utilizes the mode-matching technique to model and analyze a trifurcated waveguide that includes impedance discontinuities. Additionally, it analyzes the behaviors of the scattered field in trifurcated lined ducts, both with and without impedance discontinuities. The physical structure is analyzed using a mode-matching method, and the study addresses the geometric discontinuities at the interface by matching the pressure and velocity modes using continuity conditions at the aperture. This approach enables the recasting of the differential system into a linear algebraic system that can be solved through inversion.

Precisely, the underlying problem provides a step further in generalizing the study of planar trifurcated lined ducts. The following sections make up the article: The basic waveguide structure is defined in section 2. In section 3, the mode-matching method is used to estimate the scattered field potentials in each region. The energy flux distribution in various regions is obtained in part 4 by numerically solving truncated infinite linear systems. In Section 5, numerical results are presented graphically. Section 6 summarizes the investigations.

## Formulation of boundary value problem

The study focuses on the propagation of acoustic waves in a waveguide with partitions and impedance discontinuities. In a rectangular coordinate system ($\bar{x},\bar{y}$), the waveguide can be divided into four regions defined as follows:

- Region $R_{1}:\bar{x}<\bar{0},\bar{y}<\left|\bar{a}\right|$
- Region $R_{2}:\bar{x}<\bar{0},-\bar{b}<\bar{y}<-\bar{a}$
- Region $R_{3}:\bar{x}<\bar{0},\bar{a}<\bar{y}<-\bar{b}$
- Region $R_{4}:\bar{x}>\bar{0},\bar{y}<\left|\bar{h}\right|$

Note that the bars in the variables represent the dimensional setting of those variables. The regions mentioned are filled with a compressible fluid with density *ρ* and sound speed *c*. In particular, region $R_{1}$ is bounded by rigid walls with an infinite impedance ${\bar{Z}}_{1}$, while the surfaces of regions $R_{2}$, $R_{3}$, and $R_{4}$ have finite impedances ${\bar{Z}}_{2}$, ${\bar{Z}}_{3}$, and ${\bar{Z}}_{4}$, respectively. Assuming a harmonic time dependence of $e^{-i\omega \bar{t}}$, where *ω* = *ck* represents the radian frequency with *k* being the wave number, the surface impedance $\bar{Z}j$ can be expressed in terms of the time-independent fluid potential $\bar{\varphi }j$, as mentioned in reference [31], that is

$$\begin{array}{c} \{\bar{n}\cdot \bar{\nabla }-i\rho \omega {\bar{Z}}_{j}^{-1}\}{\bar{\varphi }}_{j}=0, \end{array}$$ (1)

where *j* = 1, 2, 3, 4 is used to specify the regions $R_{j}$. The waveguide’s time-independent fluid potential $\bar{\varphi }$ is governed by the Helmholtz equation [31], which can be expressed as follows:

$$\begin{array}{c} \{\frac{\partial^{2}}{\partial {\bar{x}}^{2}}+\frac{\partial^{2}}{\partial {\bar{y}}^{2}}+k^{2}\}{\bar{\varphi }}_{j}=0. \end{array}$$ (2)

**Fig 1:**
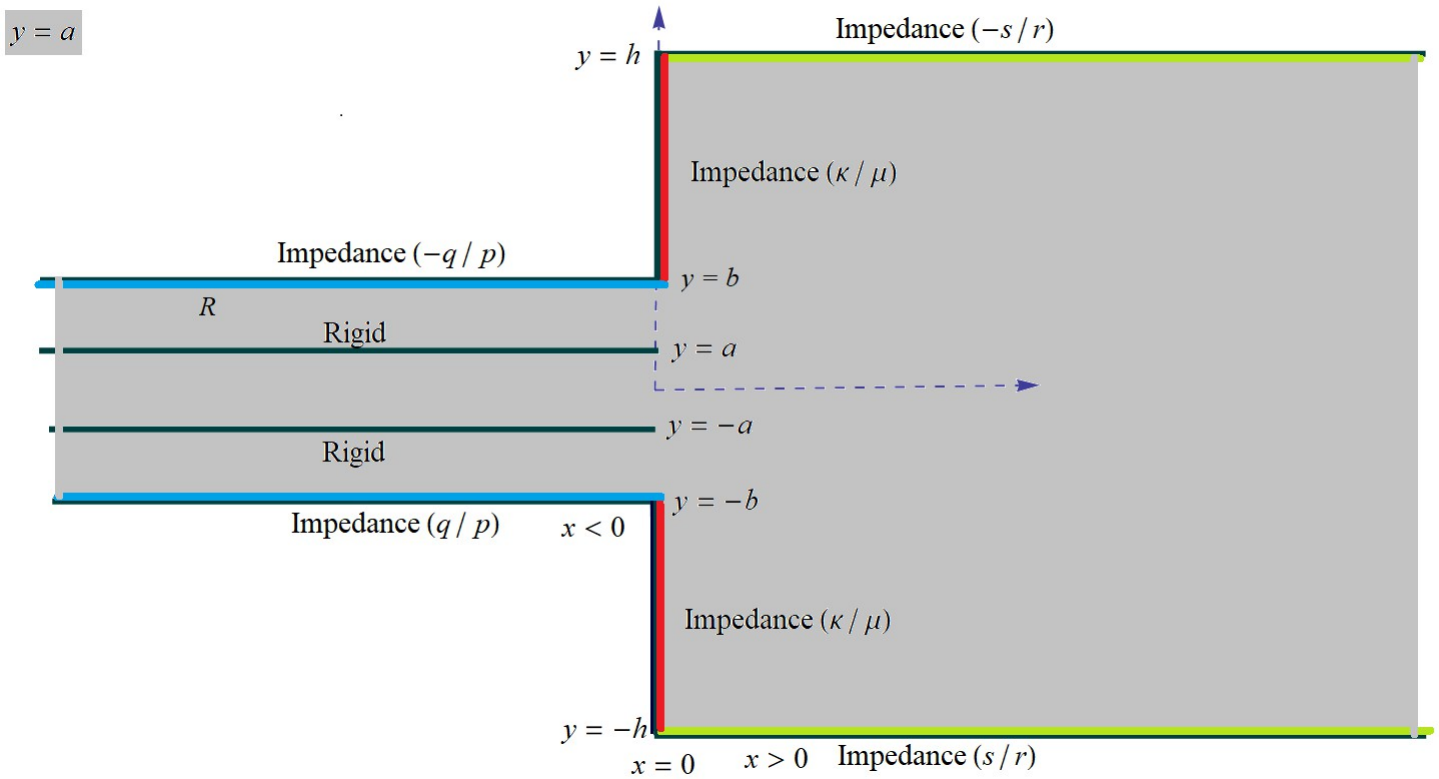
The schematic diagram of the model.

The governing boundary value problem is non-dimensionalized using the length scale *k*^−1^ and time scale *ω*^−1^ such that $x=k\bar{x},y=k\bar{y}$ and $\varphi_{j}=k^{2}\omega^{-1}{\bar{\varphi }}_{j}$. The problem’s dimensionless form is depicted in Fig 1, illustrating the schematic configuration. The non-dimensional problem incorporates Helmholtz’s equation with a unit wave number, which can be expressed as follows:

$$\begin{array}{c} (\nabla^{2}+1)\varphi_{j}\left(x,y\right)=0, \end{array}$$ (3)

where $\varphi_{j}=\omega^{-1}k^{2}{\bar{\varphi }}_{j}.$ The dimensionless form of boundary conditions are

- Rigid conditions ($Z_{1}=\rho c{\bar{Z}}_{1}=\infty$)
  $$\begin{array}{c} \frac{\partial \varphi_{1}}{\partial y}=0,\;\;\;\;\;y=\pm a, \end{array}$$ (4)
- At *y* = −*b*, the specific impedance in dimensionless form is $Z_{2}=\rho c{\bar{Z}}_{2}=q/p$, which yield condition
  $$\begin{array}{c} p\varphi_{2}-q\frac{\partial \varphi_{2}}{\partial y}=0,\;\;\;\;\;\;\;y=-b, \end{array}$$ (5)
- At *y* = *b*, the specific impedance in dimensionless form $Z_{3}=\rho c{\bar{Z}}_{3}=-q/p$ leads to
  $$\begin{array}{c} p\varphi_{3}+q\frac{\partial \varphi_{3}}{\partial y}=0,\;\;\;\;\;\;\;y=b, \end{array}$$ (6)
- At *y* = ±*h*, the specific impedance in dimensionless form $Z_{4}=\rho c{\bar{Z}}_{4}^{\pm }=\mp s/r$ give
  $$\begin{array}{c} r\varphi_{4}\pm s\frac{\partial \varphi_{4}}{\partial y}=0,\;\;\;\;\;\;\;y=\pm h, \end{array}$$ (7)
  whereas, at *x* = 0, the dimensionless impedance along −*h* ≤ *y* ≤ −*b* and *b* ≤ *y* ≤ *h* is *κ*/*μ*, that give
  $$\begin{array}{c} \mu \varphi_{4}+\kappa \frac{\partial \varphi_{4}}{\partial x}=0,\;\;\;\;x=0,\;b\leq y\leq h\;\text{and}\;-h\leq y\leq -b. \end{array}$$ (8)

## Solution methodology: Mode-matching approach

In order to understand the scattering properties of the given structure, we utilize the mode-matching technique to solve the governing boundary value problem. This technique involves obtaining the eigenfunction expansions of the duct regions and applying the matching interface conditions to convert the differential systems into linear algebraic systems. These linear algebraic systems are then truncated and inverted. In the subsequent subsections, we will explore the specifics of the eigenfunction expansions and the properties of the eigenfunctions in more detail.

### 0.1 In region $R_{1}:\{x<0,|y|<a\}$

The acoustic region denoted as $R_{1}$ is enclosed by acoustically rigid boundaries described by Eq (4). Within this region, the propagation of sound satisfies the Helmholtz’s equation as stated in Eq (3). To solve Eq (3) under the boundary condition (4), we employ the separation of variable method. This method allows us to decompose the solution into a series of eigenfunctions. The resulting solution takes the form of an eigenfunction expansion

$$\begin{array}{c} \varphi_{1}\left(x,y\right)=e^{ix}+\sum_{n=0}^{\infty }A_{n}Y_{1n}\left(y\right)e^{-i\vartheta_{n}x}. \end{array}$$ (9)

In region $R_{1}$, the eigenfunction is represented as $Y_{1n}\left(y\right)=\text{cos}\left[\tau_{n}\left(y+a\right)\right]$. Here, *ϑ*_*n*_ denotes the wave number of the *n*-th mode and is defined as $\vartheta_{n}=\sqrt{1-\tau_{n}^{2}}$. The eigenvalues *τ*_*n*_ satisfy the dispersion relation described by the following equation:

$$\begin{array}{c} \text{sin}\;[2\tau_{n}a]=0,\;\text{for}\;n=0,1,2... \end{array}$$ (10)

In Eq (9), the first term represents the incident wave, while the second term represents the reflected field. The coefficients *A*_*n*_ in the second term represent the unknown reflected mode coefficients. Additionally, the eigenfunctions $Y_{1n}\left(y\right)$ associated with this analysis also fulfill the orthogonality relation provided by:

$$\begin{array}{c} \int_{-a}^{a}Y_{1m}\left(y\right)Y_{1n}\left(y\right)dy=a\delta_{mn}\epsilon_{m}, \end{array}$$ (11)

where, *ϵ*_*m*_ = 2 for *m* = 0 and 1 otherwise and *δ*_*mn*_ is Kronecker delta.

### 0.2 In regions $R_{2}:\{x<0,-b<y<-a\}$ and $R_{3}:\{x<0,a<y<b\}$

The upper boundary of region $R_{2}$ is defined by the rigid wall condition at *y* = −*a*, as stated in Eq (4). On the other hand, the lower boundary at *y* = −*b* is governed by the impedance wall condition specified in Eq (5). Similarly, the lower boundary of region $R_{3}$ is determined by the rigid wall condition at *y* = *a*, as given in Eq (4). The upper boundary, on the other hand, is subject to the impedance wall condition at *y* = *b*, as described in Eq (6). By solving Eq (3) while considering these boundary conditions for regions R2 and R3, the eigenfunction expansion can be expressed in the following formulations:

$$\begin{array}{c} \varphi_{2}\left(x,y\right)=\sum_{n=0}^{\infty }B_{n}Y_{2n}\left(y\right)e^{-i\varkappa_{n}x}, \end{array}$$ (12)

$$\begin{array}{c} \varphi_{3}\left(x,y\right)=\sum_{n=0}^{\infty }C_{n}Y_{3n}\left(y\right)e^{-i\varkappa_{n}x}. \end{array}$$ (13)

In this context, the eigenfunctions for regions $R_{2}$ and $R_{3}$ are given by $Y_{2n}\left(y\right)=\text{cos}\left[\lambda_{n}\left(y+a\right)\right]$ and $Y_{3n}\left(y\right)=\text{cos}\left[\lambda_{n}\left(y-a\right)\right]$ respectively. The wave number associated with the *n*^th^ mode can be mathematically represented as $\varkappa_{n}=\sqrt{1-\lambda_{n}^{2}}$, where λ_*n*_ denotes the *n*^th^ eigenvalue. For the case of mixed boundary conditions at *y* = ±*b*, the eigenvalues for *n* = 0, 1, 2, ⋯ are determined as the roots of the following dispersion relation:

$$\begin{array}{c} p\;\text{cos}\;[\lambda_{n}\left(b-a\right)]-q\lambda_{n}\;\text{sin}\;[\lambda_{n}\left(b-a\right)]=0. \end{array}$$ (14)

In their respective domains, the eigenfunctions $Y_{2n}\left(y\right)$ and $Y_{3n}\left(y\right)$ are orthogonal to each other. This orthogonality is expressed through the following relations:

$$\begin{array}{c} \int_{-b}^{-a}Y_{2m}\left(y\right)Y_{2n}\left(y\right)dy=\int_{a}^{b}Y_{3m}\left(y\right)Y_{3n}\left(y\right)dy=F_{n}\delta_{mn}, \end{array}$$ (15)

where

$$\begin{array}{c} F_{n}=\frac{2\left(b-a\right)\lambda_{n}+\text{sin}\;[2\left(b-a\right)\lambda_{n}]}{4\lambda_{n}}. \end{array}$$ (16)

### 0.3 In region $R_{4}:\{x>0,|y|<h\}$

The region $R_{4}$ is bounded by impedance type conditions at *y* = ±*h*, as specified in Eq (6). Within this region, the eigenfunction expansion for the propagation of sound waves can be obtained by solving Eq (3) while considering the boundary conditions given in Eq (7). This allows for a comprehensive understanding of the behavior of sound waves within this region in term of following expansion

$$\begin{array}{c} \varphi_{4}\left(x,y\right)=\sum_{n=0}^{\infty }D_{n}Y_{4n}\left(y\right)e^{i\varsigma_{n}x}. \end{array}$$ (17)

Here $Y_{4n}\left(y\right)=r\;\text{sin}\;[\gamma_{n}\left(y+h\right)]+s\gamma_{n}\;\text{cos}\;[\gamma_{n}\left(y+h\right)]$ expresses the eigenfunction of a mode having mode wave number $\varsigma_{n}=\sqrt{1-\gamma_{n}^{2}}$ in which *γ*_*n*_ are the eigenvalues. These eigenvalues are the roots of following dispersion relation:

$$\begin{array}{c} (r^{2}-s^{2}\gamma_{n}^{2})\;\text{sin}\;(2\gamma_{n}h)+2rs\gamma_{n}\;\text{cos}\;(2\gamma_{n}h)=0. \end{array}$$ (18)

Moreover, the eigenfunctions $Y_{4n}\left(y\right)$ satisfy the orthogonality relation

$$\begin{array}{c} \int_{-h}^{h}Y_{4m}\left(y\right)Y_{4n}\left(y\right)dy=G_{n}\delta_{mn}, \end{array}$$ (19)

where

$$\begin{array}{c} G_{n}=\frac{1}{4\gamma_{n}}\{2\gamma_{n}\left(rs+2h\left(r^{2}+s^{2}\gamma_{n}^{2}\right)-rs\;\text{cos}\;(4h\gamma_{n})\right)+\left(-r^{2}+s^{2}\gamma_{n}^{2}\right)\;\text{sin}\;(4h\gamma_{n})\}. \end{array}$$ (20)

Note that with impedance conditions, the roots of dispersion relations (14) and (18) must be found numerically and must be arranged in accordance with the properties as given in [14]. Furthermore, the coefficients {*A*_*n*_, *B*_*n*_, *C*_*n*_, *D*_*n*_} are unknowns. To find these unknowns, we use matching interface conditions.

### 0.4 Matching interface conditions

Regarding the conditions governing interface matching, our focus is on ensuring the matching of pressure and normal velocity modes at the interface. To accurately capture the scattering response in the presence of impedance variations and geometric discontinuities, it is crucial to carefully consider the interface conditions. The literature provides several formulations of such conditions, as demonstrated by [14]. Our specific attention is directed towards the aperture located at the interface, precisely at *x* = 0, where achieving consistency in pressure values across different regions is of paramount importance. Simultaneously, we integrate impedance discontinuities into the matching conditions for the velocity modes. We adopt the approach of maintaining the continuity of pressure modes, normalized with respect to the eigenfunctions of regions $R_{1}$, $R_{2}$, and $R_{3}$, thereby providing a comprehensive framework for an accurate representation of the system.

$$\begin{array}{c} \int_{-a}^{a}Y_{1m}\left(y\right)\varphi_{1}(0,y)dy=\int_{-a}^{a}Y_{1m}\left(y\right)\varphi_{4}(0,y)dy, \end{array}$$ (21)

$$\begin{array}{c} \int_{-b}^{-a}Y_{2m}\left(y\right)\varphi_{2}(0,y)dy=\int_{-b}^{-a}Y_{2m}\left(y\right)\varphi_{4}(0,y)dy, \end{array}$$ (22)

$$\begin{array}{c} \int_{a}^{b}Y_{3m}\left(y\right)\varphi_{3}(0,y)dy=\int_{a}^{b}Y_{3m}\left(y\right)\varphi_{4}(0,y)dy, \end{array}$$ (23)

By incorporating the eigenfunction expansions (9), (12), (13) and (17) into Eqs (21)–(23), and subsequently solving the resultant equations with the assistance of the orthogonality relations outlined in (11) and (15), and following certain mathematical rearrangements, we derive the explicit expression for the scattering amplitudes as follows:

- For region $R_{1}$, we get
  $$\begin{array}{c} A_{m}=\frac{-2\delta_{m0}}{\varepsilon_{m}}+\frac{1}{a\varepsilon_{m}}\sum_{n=0}^{\infty }D_{n}R_{mn}, \end{array}$$ (24)
  where
  $$\begin{array}{c} R_{mn}=\frac{\gamma_{n}}{\tau_{m}^{2}-\gamma_{n}^{2}}\{{\left(-1\right)}^{m}H_{n}^{+}-H_{n}^{-}\}, \end{array}$$ (25)
  in which $H_{n}^{\pm }=r\;\text{cos}\;[\gamma_{n}\left(h\pm a\right)]-s\gamma_{n}\;\text{sin}\;[\gamma_{n}\left(h\pm a\right)].$
- For region $R_{2}$, it is found that
  $$\begin{array}{c} B_{m}=\frac{1}{F_{m}}\sum_{n=0}^{\infty }D_{n}P_{mn}, \end{array}$$ (26)
  where
  $$\begin{array}{c} P_{mn}=\frac{1}{\gamma_{n}^{2}-\lambda_{m}^{2}}\{\gamma_{n}J_{n}+\gamma_{n}\;\text{cos}\;\left[\lambda_{m}\left(b-a\right)\right]K_{n}-\lambda_{m}\;\text{sin}\;\left[\lambda_{m}\left(b-a\right)\right]L_{n}\}, \end{array}$$ (27)
  in which *J*_*n*_ = −*r* cos [*γ*_*n*_(*h* − *a*)] + *sγ*_*n*_ sin [*γ*_*n*_(*h* − *a*)], *K*_*n*_ = *r* cos [*γ*_*n*_(*h* − *b*)] − *sγ*_*n*_ sin [*γ*_*n*_(*h* − *b*)] and *L*_*n*_ = *r* sin [*γ*_*n*_(*h* − *b*)] + *sγ*_*n*_ cos [*γ*_*n*_(*h* − *b*)].
- For region $R_{3}$, we achieve
  $$\begin{array}{c} C_{m}=\frac{1}{F_{m}}\sum_{n=0}^{\infty }D_{n}Q_{mn}, \end{array}$$ (28)
  where
  $$\begin{array}{c} Q_{mn}=\frac{1}{\gamma_{n}^{2}-\lambda_{m}^{2}}\{\gamma_{n}M_{n}+\gamma_{n}\;\text{cos}\left[\lambda_{m}\left(b-a\right)\right]N_{n}-\lambda_{m}\;\text{sin}\left[\lambda_{m}\left(b-a\right)\right]T_{n}\}, \end{array}$$ (29)
  with *M*_*n*_ = *r* cos [*γ*_*n*_(*h* + *a*)] − *sγ*_*n*_ sin [*γ*_*n*_(*h* + *a*)], *N*_*n*_ = −*r* cos [*γ*_*n*_(*h* + *b*)] + *sγ*_*n*_ sin [*γ*_*n*_(*h* + *b*)] and *T*_*n*_ = *r* sin [*γ*_*n*_(*h* + *b*)] + *sγ*_*n*_ cos [*γ*_*n*_(*h* + *b*)].

At the interface, we utilize the matching condition of normal velocities to determine the unidentified coefficient for region R4. Normalizing these conditions with respect to the eigenfunctions of region R4 results in:

$$\begin{array}{c} \int_{-h}^{h}Y_{4m}\left(y\right)\varphi_{4x}(0,y)dy=-\frac{\mu }{\kappa }\int_{-h}^{-b}Y_{4m}\left(y\right)\varphi_{4}(0,y)dy+\int_{-b}^{-a}Y_{4m}\left(y\right)\varphi_{2x}(0,y)dy\\ +\int_{-a}^{a}Y_{4m}\left(y\right)\varphi_{1x}(0,y)dy+\int_{a}^{b}Y_{4m}\left(y\right)\varphi_{3x}(0,y)dy-\frac{\mu }{\kappa }\int_{b}^{h}Y_{4m}\left(y\right)\varphi_{4}(0,y)dy \end{array}$$ (30)

Utilizing the fluid potentials provided in (9), (12), (13) and (17) in Eq (30), and subsequently applying the orthogonality relation (19), one can obtain the explicit formulation of scattering amplitudes of region R4 through some mathematical rearrangements:

$$\begin{array}{c} D_{m}\varsigma_{m}G_{m}=R_{0m}-\sum_{n=0}^{\infty }A_{n}\vartheta_{n}R_{nm}-\sum_{n=0}^{\infty }B_{n}\varkappa_{n}P_{nm}-\sum_{n=0}^{\infty }C_{n}\varkappa_{n}Q_{nm}\\ +\frac{i\mu }{\kappa }\sum_{n=0}^{\infty }D_{n}(S_{mn}+H_{mn}), \end{array}$$ (31)

where

$$\begin{array}{c} S_{mn}=\int_{b}^{h}Y_{4m}\left(y\right)Y_{4n}\left(y\right)dy\;\text{and}\;H_{mn}=\int_{-h}^{-b}Y_{4m}\left(y\right)Y_{4n}\left(y\right)dy. \end{array}$$

By applying Eqs (24), (26) and (28) into Eq (31), we obtain a linear algebraic system with unknowns *D*_*m*_, where *m* = 0, 1, 2, ⋯. To determine these unknowns, the system is truncated and then inverted. Once the values for *D*_*m*_ are determined, the quantities *A*_*n*_, *B*_*n*_, *C*_*n*_ can be easily calculated using Eqs (24), (26) and (28). It is important to note that the system for the rigid discontinuities at *x* = 0 can be derived from Eq (31) by setting *μ* = 0.

## Energy flux

Energy flux or power forms the basis for quantifying the distribution of energy across various aspects of the guiding structure, enabling a comprehensive understanding of its scattering behavior. The formulas for radiated energy flux, reflection, and transmission can be determined by applying the definition provided in [14], which is:

$$\begin{array}{c} Energy\;flux=\frac{1}{2}Re\{i\int_{\Omega }\varphi {\left(\frac{\partial \varphi }{\partial x}\right)}^{*}dy\}, \end{array}$$ (32)

where superscript asterisk (*) denotes the complex conjugate. By substituting the incident field *e*^*ix*^ from Eq (9) into Eq (32), we can determine the incidence power as $P_{inc}=a$. Similarly, by substituting the reflecting and transmitting fields into Eq (32), we can calculate the power or energy flux in the duct sections Rj as follows:

$$\begin{array}{c} P_{1}=-\frac{1}{2}Re\{\sum_{n=o}^{\infty }{\left|A_{n}\right|}^{2}\vartheta_{n}\varepsilon_{n}\}, \end{array}$$ (33)

$$\begin{array}{c} P_{2}=-\frac{1}{2a}Re\{\sum_{n=o}^{\infty }{\left|B_{n}\right|}^{2}\varkappa_{n}F_{n}\}, \end{array}$$ (34)

$$\begin{array}{c} P_{3}=-\frac{1}{2a}Re\{\sum_{n=o}^{\infty }{\left|C_{n}\right|}^{2}\varkappa_{n}F_{n}\}, \end{array}$$ (35)

and

$$\begin{array}{c} P_{4}=\frac{1}{2a}Re\{\sum_{n=o}^{\infty }{\left|D_{n}\right|}^{2}\varsigma_{n}G_{n}\}. \end{array}$$ (36)

Note that the negative sign in (33)–(35) indicates that the powers are propagating in negative direction. The energy conservation law can be established by equating the powers propagating in negative and positive directions, that gives

$$\begin{array}{c} P_{inc}+P_{1}+P_{2}+P_{3}=P_{4}. \end{array}$$ (37)

For analytical purposes, $P_{inc}$ is adjusted at unity, which is achieved on dividing (37) by *a* that is;

$$\begin{array}{c} 1=E_{1}+E_{2}+E_{3}+E_{4}, \end{array}$$ (38)

where, $E_{j}=-P_{j}/a$ for *j* = 1, 2, 3 and $E_{4}=P_{4}/a$. It’s worth noting that Eq (38) is recognized as the conserved power identity, rooted in the principle of energy conservation. This identity implies that if one unit of power is input into the system, it will be equivalent to the combined sum of reflected and transmitted powers.

## Numerical results and discussions

To solve the linear algebraic system presented in Eq (31), a numerical approach is employed by setting *m* = *n* = 0, 1, 2, ⋯. This enables us to obtain the truncated amplitudes. In the numerical computations, a fluid density of *ρ* = 1.2043 kgm^−3^ and a sound speed of *c* = 343ms^−1^ are considered.

Before delving into the scattering properties of the structure, it is important to evaluate the accuracy of the truncated solution. This can be achieved by numerically reconstructing the matching conditions and conservation law using the truncated form of the solution. To assess the accuracy, specific values are assigned to the parameters involved. In this case, we set $\bar{a}=0.24\text{m}$, $\bar{b}=3\bar{a}$, $\bar{h}=5\bar{a}$, *p* = *q* = *μ* = *κ* = *r* = *s* = 1, and *N* = 120, thereby establishing the structure setting and impedance. Furthermore, a frequency of *f* = 230Hz is chosen as the operating frequency.

**Fig 2:**
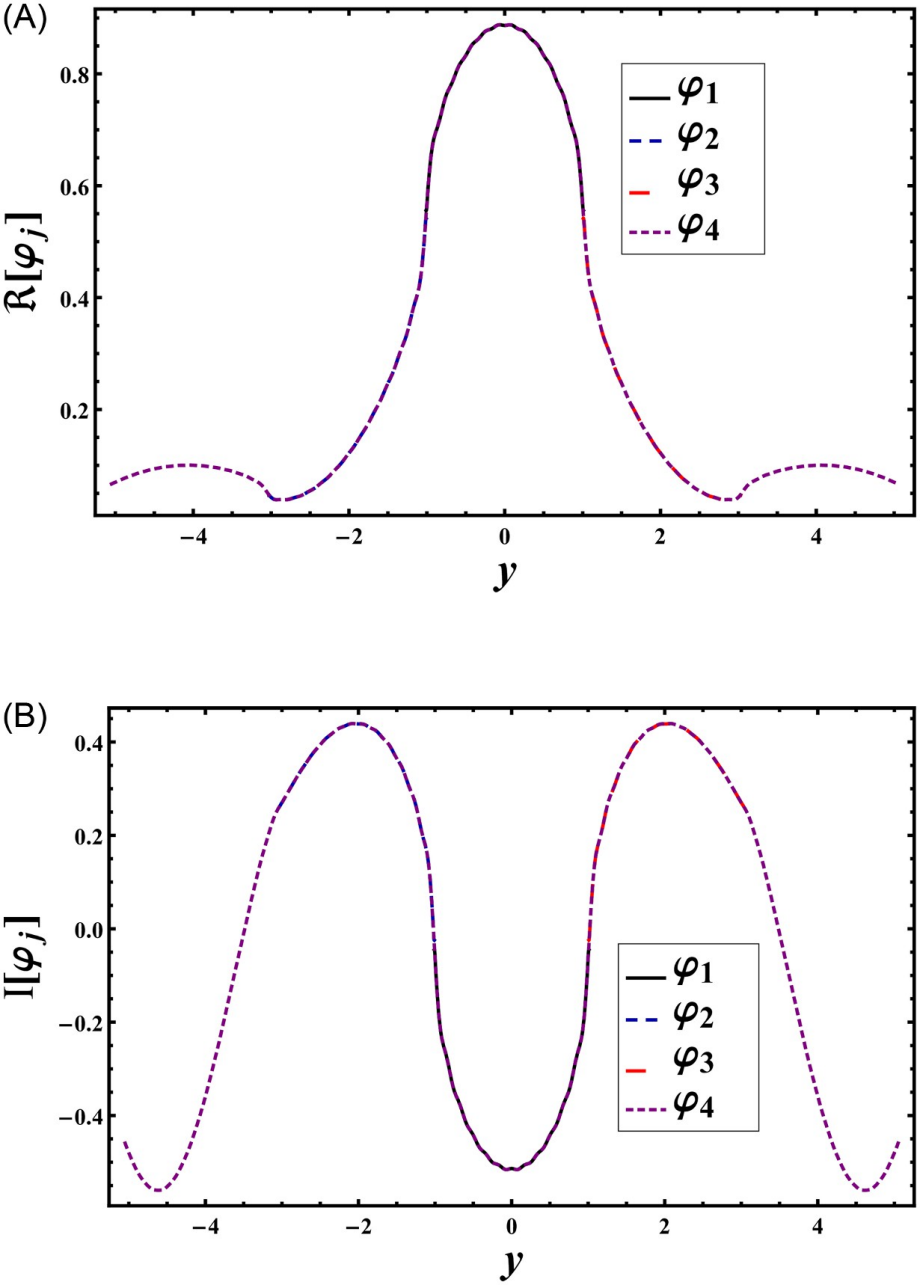
Pressure is plotted versus duct height for $\bar{a}=0.24\text{m}$, $\bar{b}=3\bar{a}$, $\bar{h}=5\bar{a},$ *p* = *q* = *μ* = *κ* = *r* = *s* = 1 and *N* = 120, where (A) the real parts and (B) the imaginary parts.

**Fig 3:**
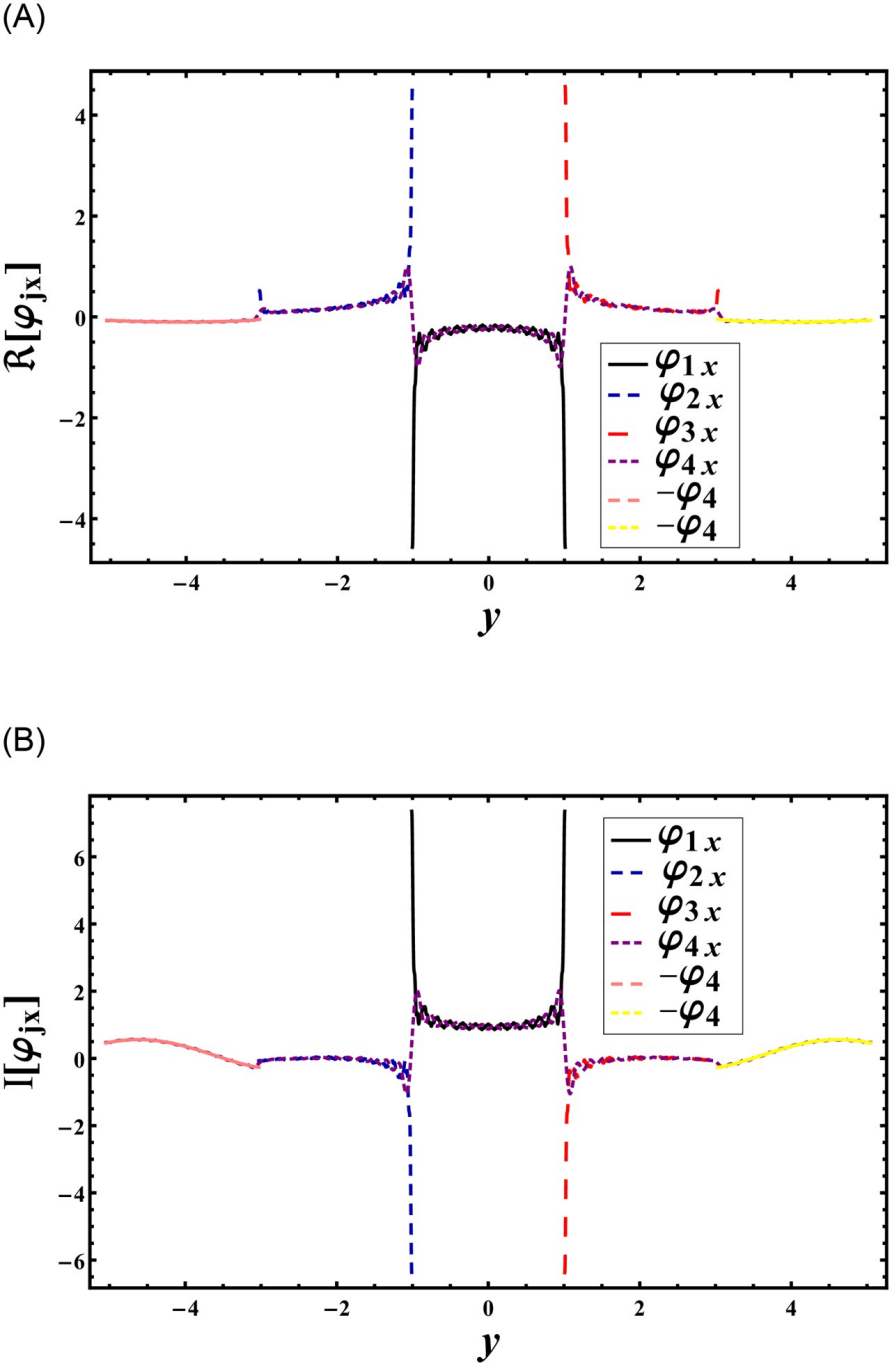
Normal velocity is plotted versus duct height for $\bar{a}=0.24\text{m}$, $\bar{b}=3\bar{a}$, $\bar{h}=5\bar{a},$ *p* = *q* = *μ* = *κ* = *r* = *s* = 1 and *N* = 120, where (A) the real parts and (B) the imaginary parts.

Figs 2 and 3 provide visual representations of the matching conditions for pressures and normal velocities at the interface *x* = 0 with respect to *y*. Fig 2 demonstrates that the real and imaginary parts of pressures, denoted as *φ*_4_(0, *y*), precisely align with *φ*_1_(0, *y*) within the domain |*y*| < *a*, *φ*_2_(0, *y*) within the domain −*b* < *y* < −*a*, and *φ*_3_(0, *y*) within the domain *a* < *y* < *b*. Similarly, Fig 3 illustrates that the real and imaginary components of the normal velocity, *φ*_4*x*_(0, *y*), perfectly match at the aperture with *φ*_1*x*_(0, *y*) in the domain |*y*| < *a*, *φ*_2*x*_(0, *y*) in the domain −*b* < *y* < −*a*, and *φ*_3*x*_(0, *y*) in the domain *a* < *y* < *b*. Additionally, along the impedance discontinuities, the real and imaginary components of the normal velocity, *φ*_4*x*_(0, *y*), coincide with −*φ*_4_ for *κ* = *μ* = 1 within the range −*h* < *y* < −*b* and *b* < *y* < *h*. This matching aligns with the assumptions made in Eqs (21)–(23) and (30), indicating that the truncated amplitudes have adequately converged. Note that to analyze how changes in the geometric proportions of the duct structure affect the transmission and absorption of sound waves some specific parametric setting is used. The parameter settings, such as $\bar{b}=3\bar{a}$, were chosen to simulate realistic scenarios encountered in duct structures, where variations in dimensions are common. This parameter setting aligns with previous studies in the literature, for instance see [16], enabling comparisons and ensuring consistency in methodology. Furthermore, our aim is not only to model specific real-world scenarios but also to explore a range of plausible configurations to elucidate general trends and behaviors. Therefore, while the chosen parameters may not correspond directly to a particular practical problem, they serve the purpose of elucidating the physical behavior of the system under varied conditions, contributing to a deeper understanding of acoustic phenomena in duct structures.

**Fig 4:**
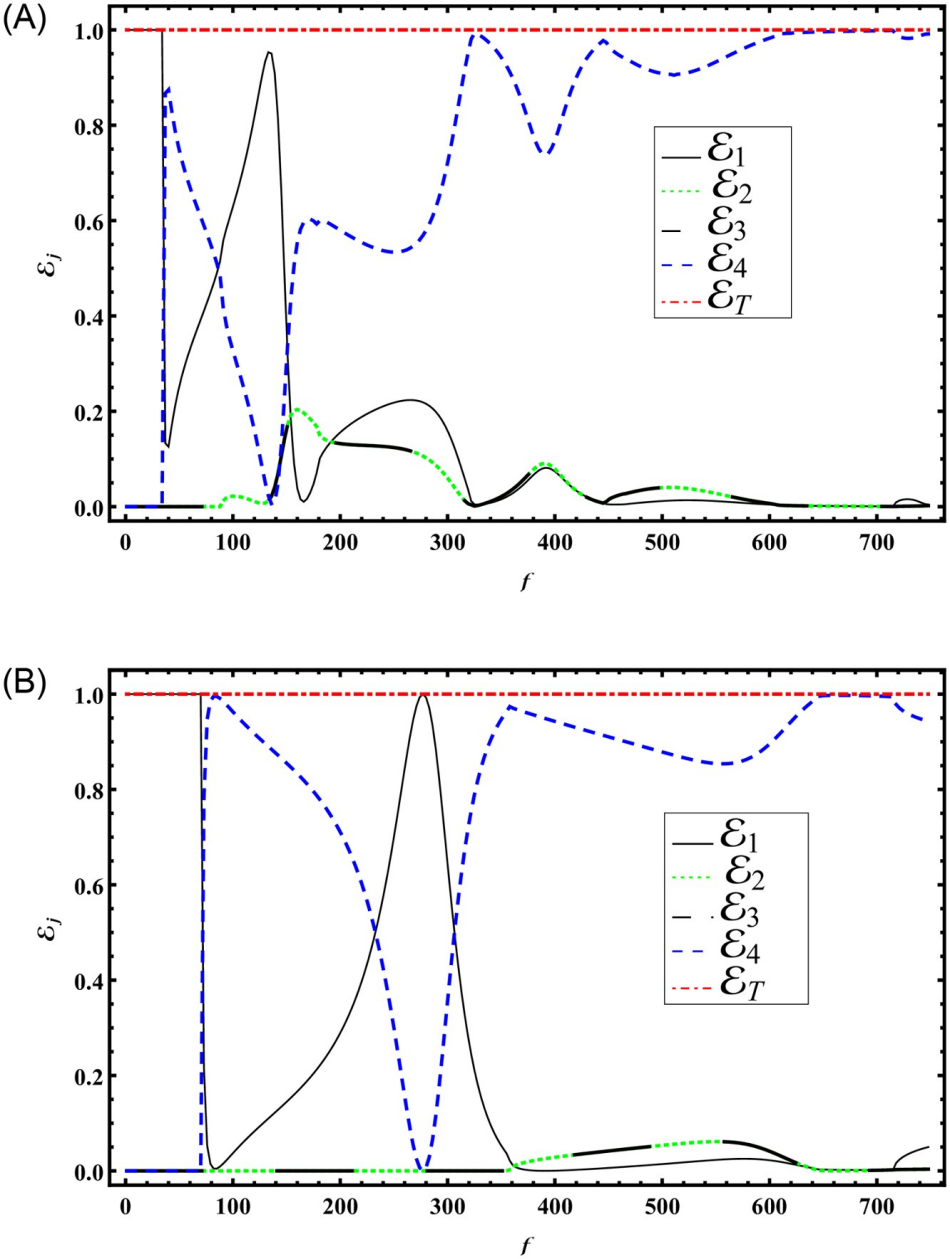
Energies versus frequency with $\bar{a}=0.24\text{m}$, $\bar{h}=5\bar{a}$, *r* = *s* = *p* = *q* = 1 for (A) $\bar{b}=3\bar{a}$ (B) $\bar{b}=\frac{3\bar{a}}{2}$.

**Fig 5:**
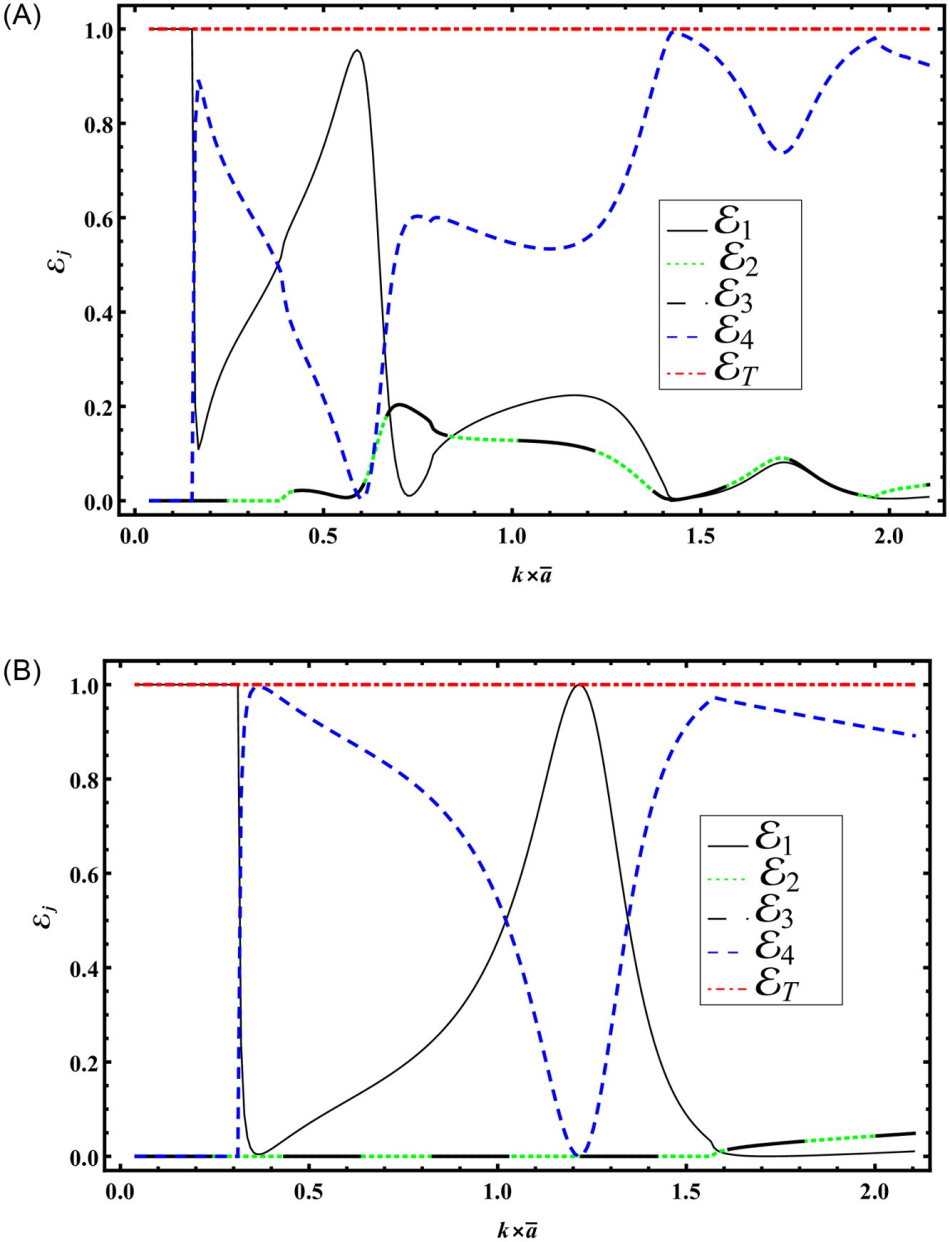
Energies versus *a* with *f* = 230Hz, *r* = *s* = *p* = *q* = 1 for (A) $\bar{b}=3\bar{a}$ (B) $\bar{b}=\frac{3\bar{a}}{2}$.

Figs 4 and 5 illustrate the energy propagation in the waveguide with respect to frequency (*f*) and the variation in symmetric height discontinuities ($a=k\times \bar{a}$), respectively. To obtain the results depicted in Fig 4, we fix the height of region $R_{1}$ as $\bar{a}=0.24\text{m}$, while the impedance parameters are assumed to be *r* = *s* = *p* = *q* = 1. The dimensions of the other regions are defined in terms of $\bar{a}$. Specifically, for region $R_{4}$, the height is set to $\bar{h}=5\bar{a}$. For regions $R_{2}$ and $R_{3}$, two values of height $\bar{b}$ are considered: 1) $\bar{b}=3\bar{a}$, and 2) $\bar{b}=\frac{3\bar{a}}{2}$. The corresponding results are shown in parts (A) and (B) of Fig 4. The system is truncated with *N* = 50 terms.

From Fig 4(A) the abrupt variations against frequency are shown. These variations are caused by the occurrence cut-on modes of region $R_{1}$. These cut-on values appear at frequency values *f* = 40, 133, 166, 328, 391, 457, 523, 715Hz. Note that there are eight cut-on values appeared in the specified domain. But by decreasing the height of $R_{2}$ and $R_{3}$ to $\bar{b}=1.5\bar{a}$, the abrupt variations are decreased, see Fig 4(B). The reason behind is the lowering of the propagating modes to four. These cut-ons appear at frequencies 82, 277, 391, 709Hz. The observed abrupt variations in the transmission and reflection properties against frequency, as depicted in Fig 4(A), stem from the resonance phenomena which occurred just before every cut-on mode within region $R_{1}$. These modes, occurring eight different frequencies signify resonant conditions where the system’s response is significantly enhanced due to the alignment of wave characteristics with structural and material properties. However, by reducing the heights of regions $R_{2}$ and $R_{3}$ to $\bar{b}=1.5\bar{a}$, as illustrated in Fig 4(B), these abrupt variations diminish. This reduction is attributed to a decrease in the number of propagating modes supported by the system, leading to a smoother transition between transmission and reflection characteristics. The altered resonance spectrum, with cut-on modes now appearing at four different frequencies, underscores the sensitivity of the system to geometric parameters and highlights opportunities for optimizing its performance in practical applications.

To obtain the results depicted in Fig 5, we vary the height of region $R_{1}$ and fix the impedance parameters to be *r* = *s* = *p* = *q* = 1, the dimensions of the other regions such that $\bar{h}=5\bar{a}$. However, for regions $R_{2}$ and $R_{3}$, two values of height $\bar{b}$ are considered: 1) $\bar{b}=3\bar{a}$, and 2) $\bar{b}=\frac{3\bar{a}}{2}$. The corresponding results are shown in parts (A) and (B) of Fig 5. The system is truncated with *N* = 50 terms.

**Fig 6:**
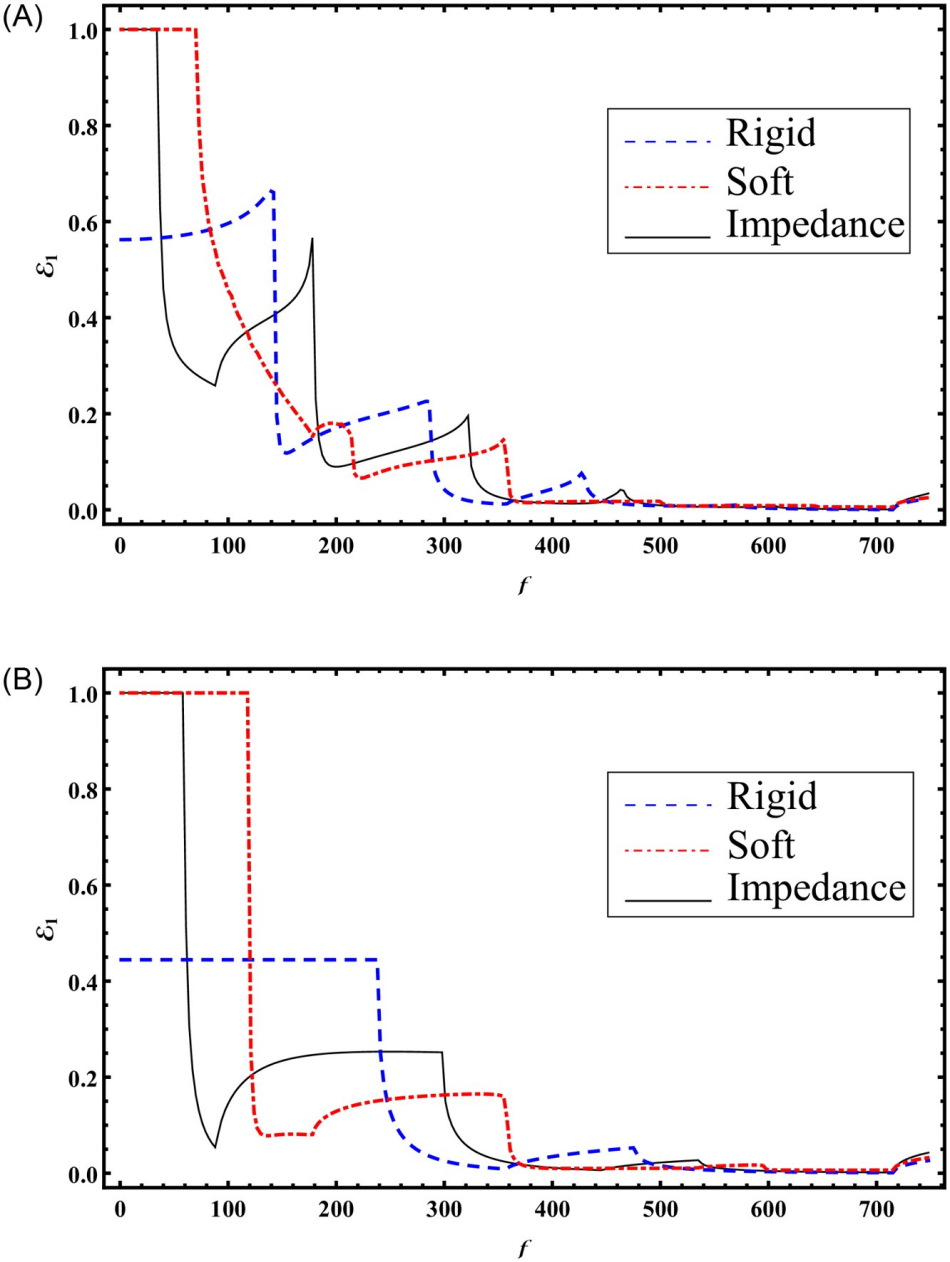
Reflected energies against frequency for rigid, soft and impedance conditions; (A) with step-discontinuities ($\bar{h}=5\bar{a}$) (B) without-discontinuities ($\bar{h}=\bar{b}$), where $\bar{b}=3\bar{a}$.

**Fig 7:**
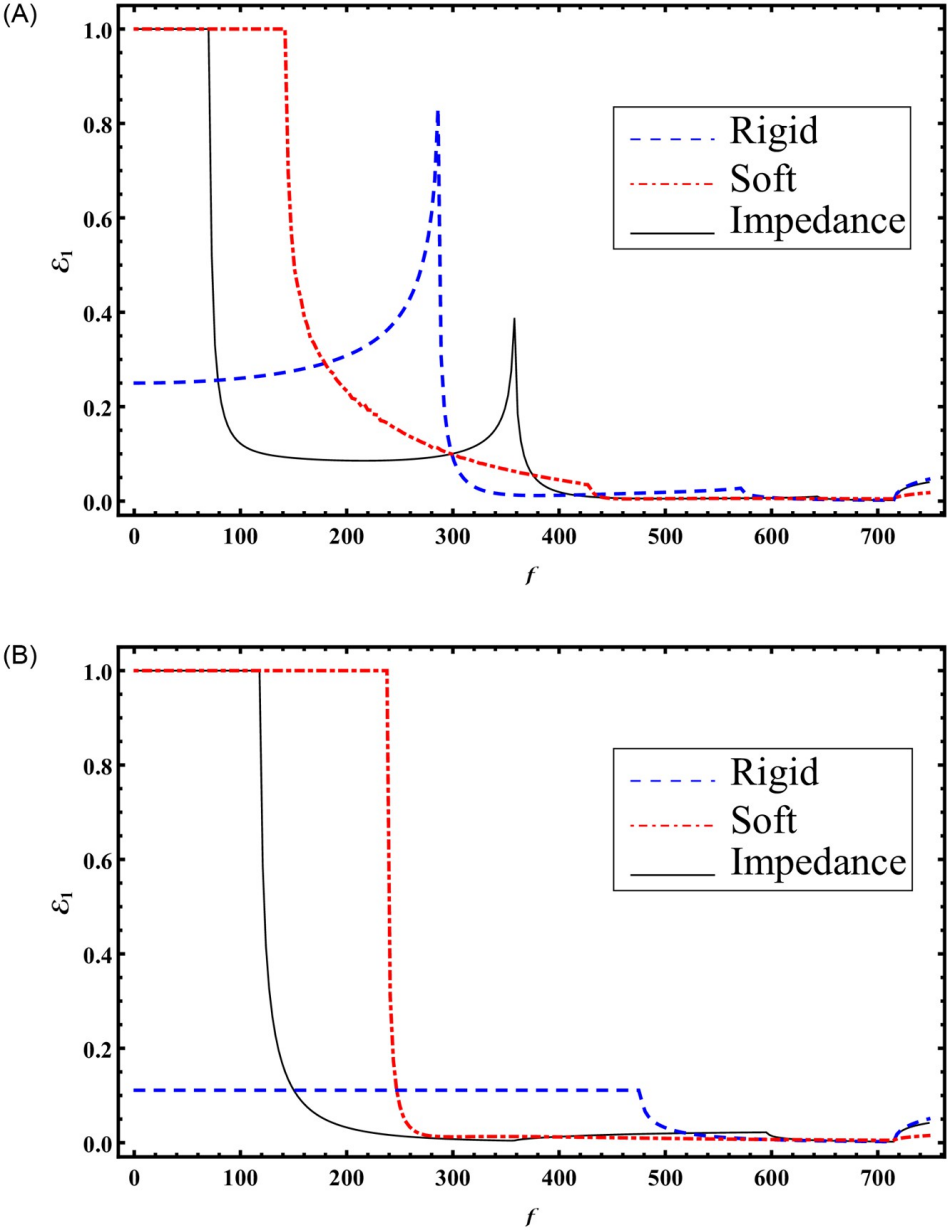
Reflected energies against frequency for rigid, soft and impedance conditions; (A) with step-discontinuities ($\bar{h}=5\bar{a}$) (B) without-discontinuities ($\bar{h}=\bar{b}$), where $\bar{b}=1.5\bar{a}$.

**Fig 8:**
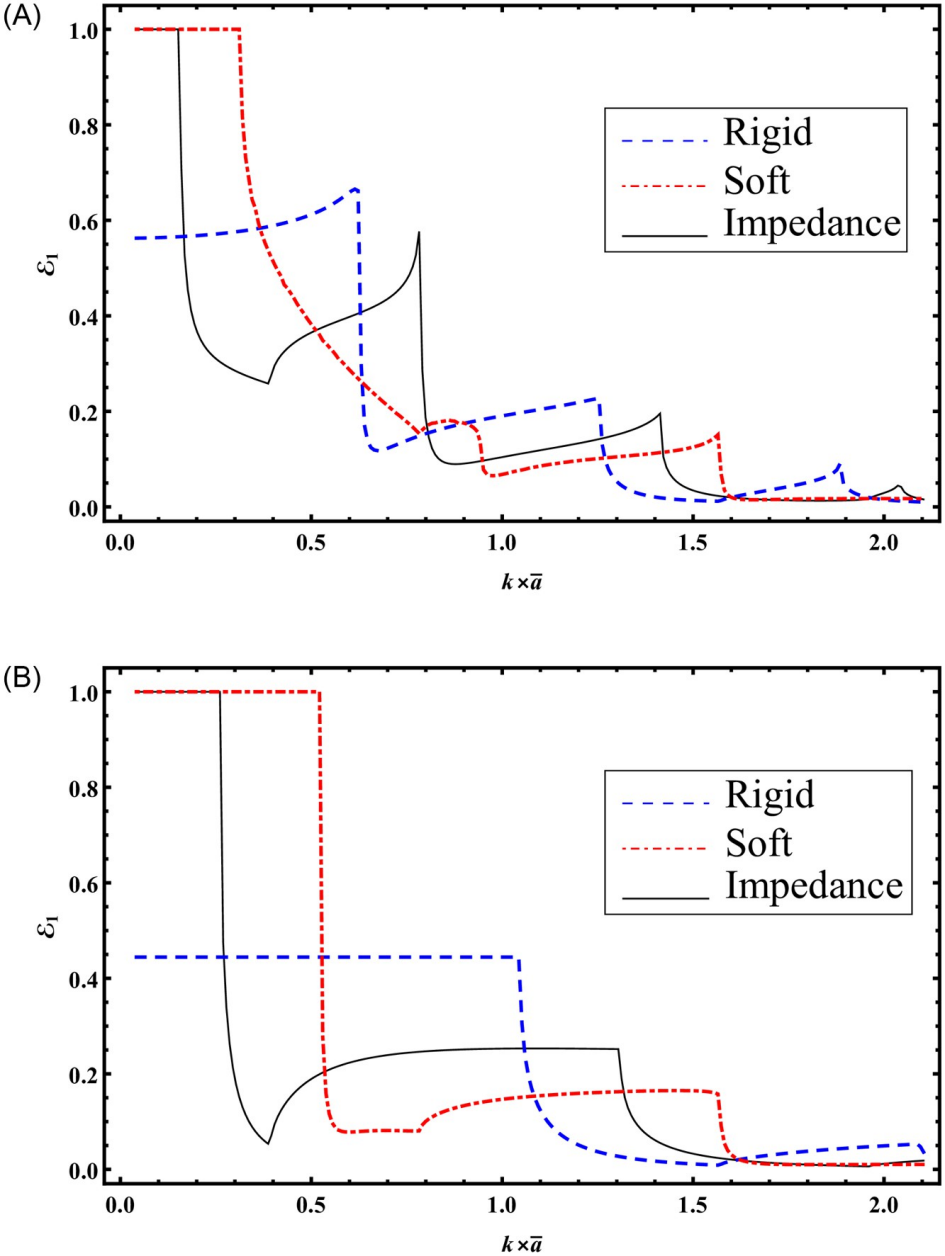
Reflected energies against $a=k\times \bar{a}$ for rigid, soft and impedance conditions; (A) with step-discontinuities ($\bar{h}=5\bar{a}$) (B) without-discontinuities ($\bar{h}=\bar{b}$), where $\bar{b}=3\bar{a}$.

**Fig 9:**
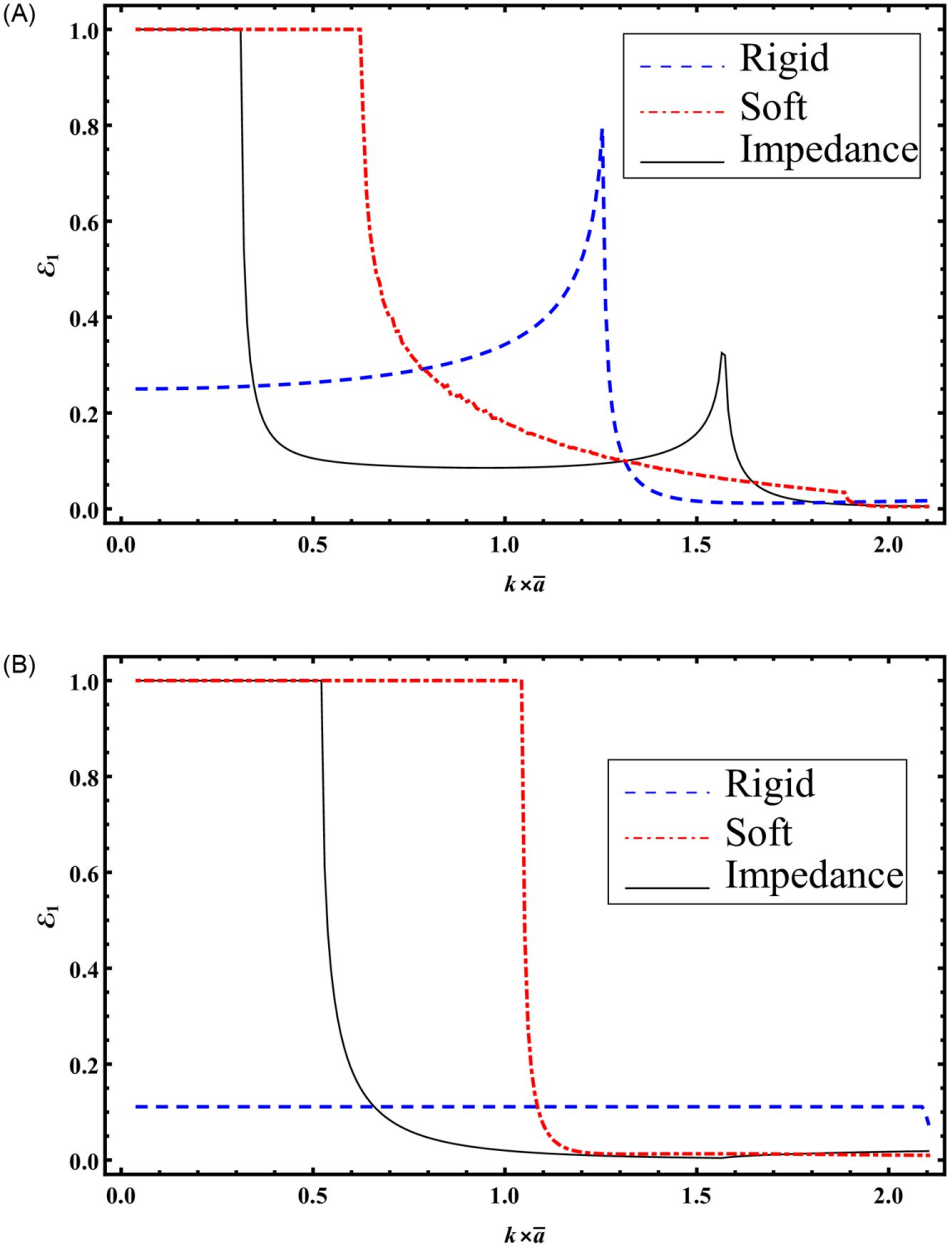
Reflected energies against $a=k\times \bar{a}$ for rigid, soft and impedance conditions; (A) with step-discontinuities ($\bar{h}=5\bar{a}$) (B) without-discontinuities ($\bar{h}=\bar{b}$), where $\bar{b}=1.5\bar{a}$.

It is noted that seven cut-on modes occur at $\bar{a}=0.168283$, 0.588991, 0.723618, 1.16115, 1.43882, 1.71649, 2.00257 m when $\bar{b}=3\bar{a}$ and these decrease to three cut-on modes occur at $\bar{a}=0.370223$, 1.22847, 1.71649 m for $\bar{b}=1.5\bar{a}$. These modes are the main cause of the abrupt variations in the scattering graphs depicted in Fig 5(A) and 5(B). Moreover, from Figs 4 and 5 we have seen that the sum of reflecting and transmitting powers is unity as is considered in (38). This further justifies the accuracy of truncated amplitudes. Now to see how the change of surface conditions impact the scattering characteristics of bifurcated structure, Figs 6–9 are shown. Three types of surface conditions are assumed: 1) Rigid conditions (*p* = *r* = *μ* = 0); 2) Soft conditions (*q* = *s* = *κ* = 0); and 3) Impedance conditions (*p* = *r* = *μ* = *q* = *s* = *κ* = 1). Further, the structure is assumed to have structural discontinuities and do not contain structural discontinuities. The results with structural discontinuities are displayed in parts (A) of Figs 6–9, and the results without structural discontinuities are displayed in parts (B) of Figs 6–9.

In Figs 6 and 7, the reflected powers are shown against the frequency with $\bar{b}=3\bar{a}$ and $\bar{b}=1.5\bar{a}$, respectively, where, $\bar{a}=0.24\text{m}$. The system is truncated by considering *N* = 50 terms. Note that to get Figs 6(A) and 7(A), we have considered $\bar{h}=5\bar{a}$ while to compute Figs 6(B) and 7(B) we have taken $\bar{h}=\bar{b}$. From the graphs shown in Figs 6 and 7, it can be seen that by changing the surface conditions form reflecting powers are changed. Therefore, one may see that by changing the surface conditions the scattering behavior can be optimized. Moreover, by using the step discontinuities the reflected powers are varied. The reason behind is the participation of more number of propagating modes with step-discontinuities involving structure as compared with the planar structure. More specifically, with step discontinuities involving settings Figs 6(A) and 7(A), the number of cut-modes respectively are 6 and 3 which appear at frequencies (88, 178, 322, 463, 607, 715) Hz and (355, 595, 715)Hz. On the other hand, with planar setting when $\bar{h}=\bar{b}$ the cut-on modes for Figs 6(B) and 7(B) are 4 and 2 which respectively appear at (88, 298, 535, 715)Hz and (358, 715)Hz.

In Figs 8 and 9, the reflected powers are depicted against $a=k\bar{a}$ with $\bar{b}=3\bar{a}$ and $\bar{b}=\frac{3\bar{a}}{2}$, respectively, at a frequency of *f* = 230Hz. The system is truncated with *N* = 50 terms. It is important to note that Figs 8(A) and 9(A) were generated with $\bar{h}=5\bar{a}$, while Figs 8(B) and 9(B) were computed using $\bar{h}=\bar{b}$. Analysis of Figs 8 and 9 reveals that altering the surface conditions leads to variations in reflecting powers, showcasing the potential for optimizing scattering behavior through such changes.

Furthermore, the introduction of step discontinuities results in varying reflected powers. This phenomenon is attributed to the involvement of a greater number of propagating modes with step discontinuities compared to a planar structure. Specifically, for Figs 8(A) and 9(A) with step discontinuities, the number of cut-modes is 6 and 3, respectively, corresponding to values of $\bar{a}$ around 0.387052, 0.782517, 0.875073, 1.41358, 1.91002, 2.03623m and 0.387052, 1.3042, 1.9605m. In contrast, with a planar setting (Figs 8(B) and 9(B)) and $\bar{h}=\bar{b}$, the cut-on modes are 3 and 1, respectively. These cut-on modes are the primary contributors to abrupt variations in scattering powers.

The aforementioned findings provide significant insights into the intricate interplay between geometric parameters, surface conditions, and scattering characteristics within bifurcated structures. Our investigation, as depicted in Fig 5, reveals that varying the height of $R_{1}$ while maintaining fixed impedance parameters leads to a notable reduction in cut-on modes from seven to three as $\bar{b}$ decreases from $3\bar{a}$ to $1.5\bar{a}$. This reduction in cut-on modes correlates with diminished abrupt variations in scattering graphs, emphasizing the sensitivity of the system to geometric changes and suggesting avenues for optimization. Transitioning to Figs 6–9, where surface conditions are systematically altered, unveils the profound impact on scattering behavior. Distinct surface conditions yield diverse reflected powers, underscoring the potential for optimizing scattering behavior through strategic adjustments. Furthermore, the introduction of step discontinuities induces significant variations in reflected powers due to the involvement of a greater number of propagating modes. Specifically, the comparison between settings with and without step discontinuities highlights the pronounced influence on cut-on modes and subsequent abrupt variations in scattering powers. Overall, these findings offer valuable insights into optimizing the scattering behavior of bifurcated structures, holding promise for practical applications across engineering and physics domains.

The surfaces within the duct regions can be designed to absorb sound effectively by selecting specific parametric settings for impedance conditions. Relevant settings from existing literature, as indicated in [16], offer valuable insights into these parametric configurations. In this article, the chosen mixed parameters correspond to [16] with *p* = *r* = *μ* = 1, *q* = *s* = *κ* = *iξ*/*k*, where *ξ* defines the specific impedance as *ξ* = *ζ* + *iη*. Here, *ζ* and *η* represent the resistive and reactive components of the surface material.

**Fig 10:**
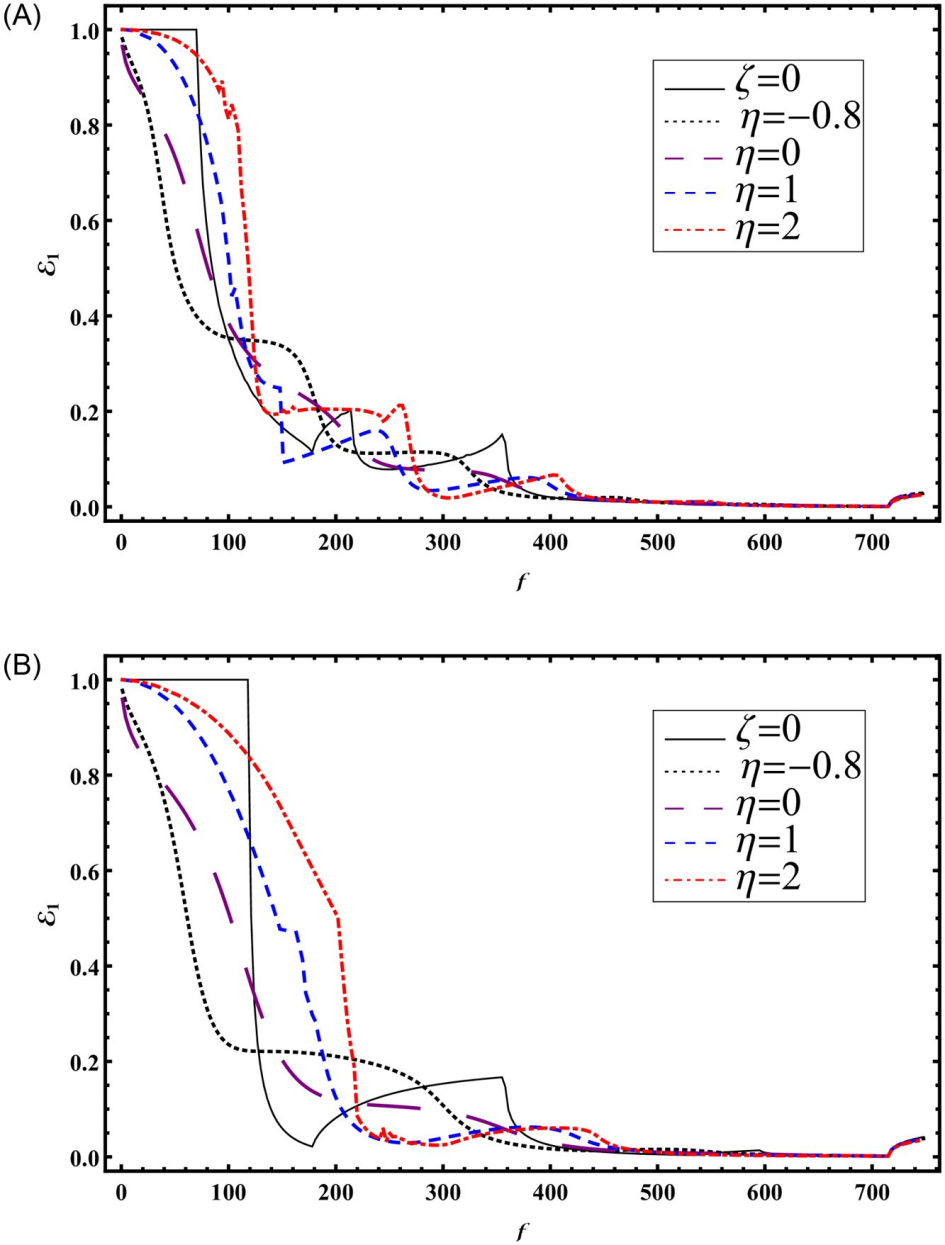
Transmitted powers against frequency with absorbent linings (A) with step-discontinuities ($\bar{h}=5\bar{a}$) (B) without-discontinuities ($\bar{h}=\bar{b}$).

**Fig 11:**
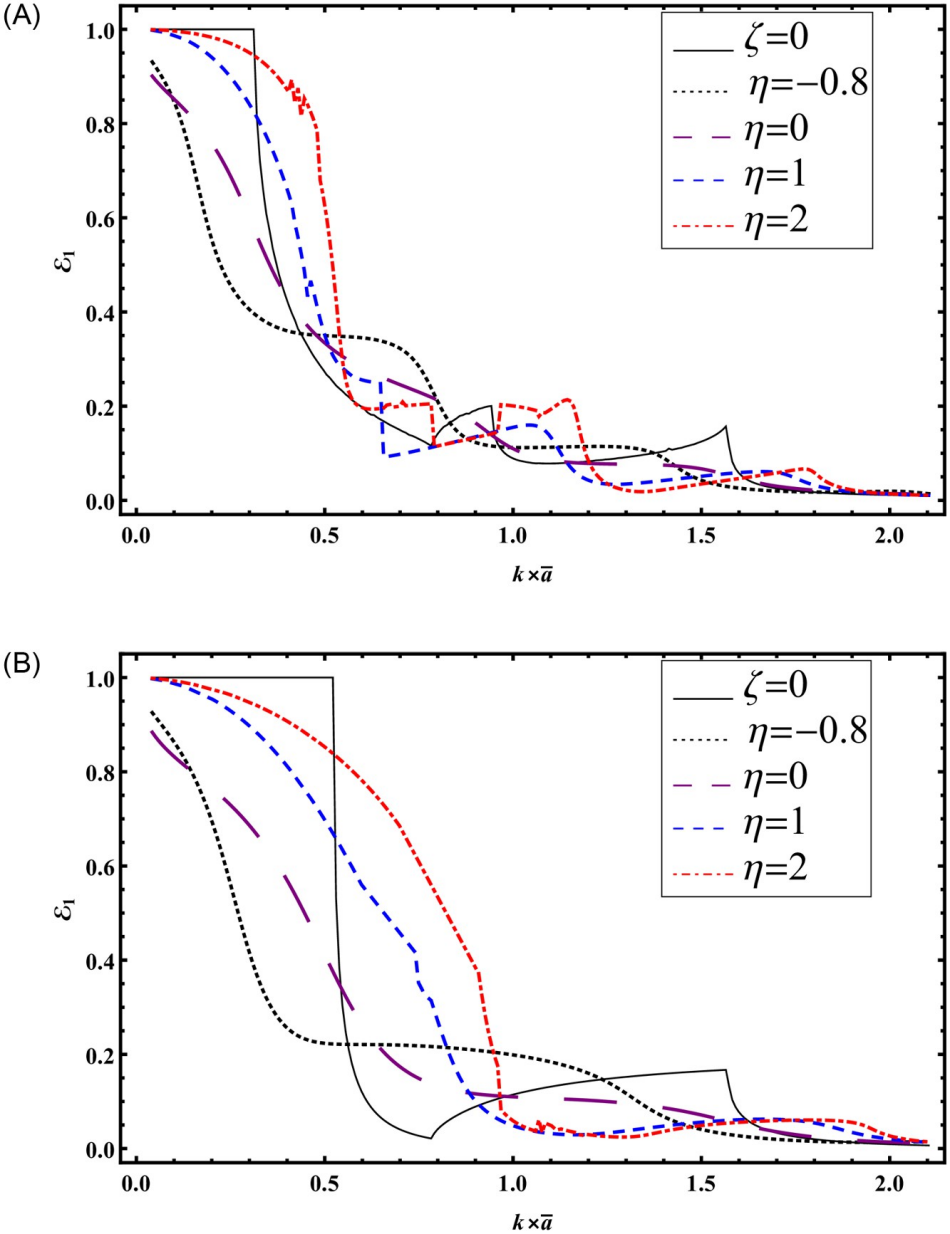
Transmitted powers against *a* with absorbent linings (A) with step-discontinuities ($\bar{h}=5\bar{a}$) (B) without-discontinuities ($\bar{h}=\bar{b}$).

To illustrate the impact of varying absorbent surfaces on transmission, Figs 10 and 11 have been generated. Results are presented against frequency and height for *ζ* = 0 and different values of *η* = −0.8, 0, 1, 2. In Fig 10, transmitted powers are plotted against frequency with $\bar{b}=3\bar{a}$ at $\bar{a}=0.24\text{m}$, while Fig 11 depicts transmitted powers against $a=k\bar{a}$ with $\bar{b}=3\bar{a}$ at a frequency of *f* = 230Hz. The system is truncated with *N* = 50 terms. Notably, for Figs 10(A) and 11(A), $\bar{h}=5\bar{a}$ was considered, while for Figs 10(B) and 11(B), $\bar{h}=\bar{b}$ was used.

Cut-on modes occurred at (178, 214, 355, 715)Hz and (178, 355, 715)Hz for Fig 10(A) and 10(B), respectively. For Fig 11(A) and 11(B), cut-on modes occurred at $\bar{a}\approx 0.782517\text{m}$, 0.942386m, 1.56503m, and $k\times \bar{a}\approx 0.782517\text{m}$, 1.56503m, respectively.

It is evident that the presence of step-discontinuities leads to more propagating cut-on modes compared to results without discontinuities. Fig 11(B) closely aligns with the findings of Rawlins [16] using the Wiener-Hopf technique, supporting the mode-matching solution computed in the presence of step-discontinuities.

Furthermore, the examination of Figs 10 and 11 provides crucial physical insights into the effect of varying absorbent surfaces on transmission characteristics within duct structures. By systematically manipulating the reactive component (*η*) while holding the resistive component constant (*ζ* = 0), these figures offer valuable insights into how different surface configurations influence the transmission of sound waves. Notably, Fig 10 presents transmitted powers plotted against frequency, offering a comprehensive view of how surface parameters impact transmission across a spectrum of frequencies, with $\bar{b}=3\bar{a}$ and $\bar{a}=0.24\text{m}$. In contrast, Fig 11 illustrates transmitted powers against height ($a=k\bar{a}$) at a fixed frequency of *f* = 230Hz, revealing the spatial variations in transmission resulting from diverse surface configurations. The identification of cut-on modes at specific frequencies in both figures further enriches our understanding. For Fig 10(A) and 10(B), where $\bar{h}=5\bar{a}$ and $\bar{h}=\bar{b}$ respectively, cut-on modes manifest at distinct frequencies, highlighting how variations in surface conditions influence the resonant behavior of the system. Similarly, in Fig 11(A) and 11(B), where the height is varied with $\bar{b}=3\bar{a}$, cut-on modes occur at different heights, demonstrating the spatial dependence of resonance within the duct structure. These findings yield critical insights for designing and optimizing sound absorption systems, empowering engineers to tailor surface parameters to achieve desired transmission characteristics in practical applications.

## Concluding remarks

The current investigation delves into the wave scattering analysis of a planar trifurcated lined duct, considering diverse boundary properties, has yielded significant insights. The study extensively explored a spectrum of mixed boundary conditions, successfully addressing the governing problem. Through the integration of eigenfunctions, orthogonality relations, and matching conditions, the initially intricate differential system underwent a transformation into a numerically solvable linear algebraic system post-truncation.

The wave scattering behavior exhibited by the trifurcated lined duct under varying conditions, including alterations in boundary properties, duct size, and impedance discontinuity is studied. The results, derived in a generalized manner, effectively recaptured existing findings for the trifurcated lined duct as a distinct case. Specifically, we successfully recovered previously established results for varied boundary properties (soft, rigid, impedance) in scenarios devoid of structural discontinuity.

Moreover, the analysis extended to the computation and scrutiny of radiated energy across all regions of the duct. A notable revelation emerged, indicating that lined ducts exhibited a reduced noise generation compared to their hard or soft counterparts. This finding underscores the practical advantage of employing lined duct configurations in situations where noise reduction is of paramount importance.

Additionally, the successful conservation of energy flux across diverse duct regions served as a validation of our algebraic approach. It confirmed the coherent propagation of cut-on duct modes within different sections, adding credibility to our methodology. Importantly, our study demonstrated that the simplified solution accurately recovered pressure and normal velocity modes, emphasizing the versatility and accuracy of our approach.
